# Low-dose aspirin and heparin treatment improves pregnancy outcome in recurrent pregnancy loss women with anti-β2-glycoprotein I/HLA-DR autoantibodies: a prospective, multicenter, observational study

**DOI:** 10.3389/fimmu.2024.1445852

**Published:** 2024-09-26

**Authors:** Kenji Tanimura, Shigeru Saito, Sayaka Tsuda, Yosuke Ono, Masashi Deguchi, Takeshi Nagamatsu, Tomoyuki Fujii, Mikiya Nakatsuka, Gen Kobashi, Hisashi Arase, Hideto Yamada

**Affiliations:** ^1^ Department of Obstetrics and Gynecology, Kobe University Graduate School of Medicine, Kobe, Japan; ^2^ Department of Obstetrics and Gynecology, University of Toyama, Toyama, Japan; ^3^ Department of Obstetrics and Gynecology, University of Yamanashi, Yamanashi, Japan; ^4^ Department of Obstetrics and Gynecology, the University of Tokyo, Tokyo, Japan; ^5^ Department of Obstetrics and Gynecology, International University of Health and Welfare Narita Hospital, Narita, Japan; ^6^ Sanno Hospital, Tokyo, Japan; ^7^ Graduate School of Health Sciences, Okayama University, Okayama, Japan; ^8^ Department of Public Health, Dokkyo Medical University School of Medicine, Tochigi, Japan; ^9^ Department of Immunochemistry, Research Institute for Microbial Disease, Osaka University, Suita, Japan; ^10^ Center for Recurrent Pregnancy Loss, Teine Keijinkai Hospital, Sapporo, Japan

**Keywords:** anti-β2-glycoprotein I/HLA-DR antibody, low-dose aspirin, pregnancy complications, recurrent pregnancy loss, treatment, unfractionated heparin

## Abstract

**Introduction:**

Anti-β2-glycoprotein I (β2GPI)/human leukocyte antigen (HLA)-DR antibodies may be a risk factor for recurrent pregnancy loss (RPL). The therapeutic modality for women with RPL and anti-β2GPI/HLA-DR antibody positivity has not been evaluated. This prospective, multicenter, observational study aimed to assess whether low-dose aspirin (LDA) and/or heparin therapies improve pregnancy outcomes in women with RPL who tested positive for anti-β2GPI/HLA-DR antibodies.

**Methods:**

Between August 2019 and December 2021, 462 women with RPL underwent anti-β2GPI/HLA-DR antibody measurements and risk assessments for RPL. Each attending physician decided the treatment modality for women with RPL who tested positive for anti-β2GPI/HLA-DR antibodies, and their pregnancy outcomes were followed up until December 2023. Finally, 47 pregnancies in 47 women with RPL and anti-β2GPI/HLA-DR antibody positivity were included in the analysis and were divided into two groups regarding whether they were treated with LDA and/or unfractionated heparin (UFH) (LDA/UFH group, n = 39) or with neither of them (non-LDA/non-UFH group, n = 8). The rates of live birth and pregnancy complications (i.e., preeclampsia and preterm delivery before 34 gestational weeks due to placental insufficiency) were compared between the two groups.

**Results:**

The live birth rate in the LDA/UFH group was higher than that in the non-LDA/non-UFH group (87.2% vs 50.0%, *p* = 0.03). The pregnancy complication rate in the LDA/UFH group was significantly lower than that in the non-LDA/non-UFH group (5.9% vs 50.0%, *p* = 0.048). Among 21 women who tested positive for anti-β2GPI/HLA-DR antibodies and had no other risk factors for RPL, the live birth rate in the LDA/UFH group (n = 14) was much higher than that in the non-LDA/non-UFH group (n = 7) (92.9% vs 42.9%, *p* = 0.03).

**Discussion:**

This study, for the first time, demonstrated that LDA and/or UFH therapies are effective in improving pregnancy outcomes in women with RPL and aβ2GPI/HLA-DR antibody positivity.

## Introduction

Recently, misfolded proteins complexed with human leukocyte antigen (HLA) class II molecules of disease-susceptible alleles, which were designated neo self-antigens, have been identified as novel autoantibody targets in patients with autoimmune diseases (1, 2). More studies have revealed that not only misfolded proteins but also DNA complexed with HLA class II molecules of disease-susceptible alleles serve as autoantibody targets, and these complexes participate in the pathogenesis of several autoimmune diseases. For example, major targets for autoantibodies include immunoglobulin (Ig) G heavy-chain/HLA-DR complexes in patients with rheumatoid arthritis, thyroid-stimulating hormone receptor/HLA-DP complexes in patients with Graves’ disease, and DNA/HLA-DR complexes in patients with systemic lupus erythematosus (2–4).

Antiphospholipid syndrome (APS) is an autoimmune disease characterized by clinical manifestations including vascular thrombosis, unexplained fetal death, recurrent pregnancy loss (RPL) and pregnancy complications together with positivity for antiphospholipid antibodies (aPLs) (5). β2-glycoprotein I (β2GPI) is a phospholipid‐binding protein and is an antigenic target for aPLs. β2GPI is expressed on several cell types associated with coagulation and with placentation (6). Previous studies have demonstrated that aPLs bind to β2GPI on trophoblasts, as well as to stromal decidual and endometrial endothelial cells. It has been shown that aPLs inhibit the differentiation and invasiveness of trophoblasts (7, 8) by inducing both trophoblast injury and apoptosis (9), and by promoting a pro-inflammatory phenotype in stromal cells (10). Additionally, aPLs have been found to inhibit angiogenesis in endometrial endothelial cells (11).

We discovered that the autoantibodies against β2GPI complexed with HLA-DR (anti-β2GPI/HLA-DR antibodies) are involved in the pathogenesis of APS with RPL and pregnancy complications (12). Anti-β2GPI/HLA-DR antibodies recognized unique epitopes on β2GPI that are not recognized by aPLs included in the laboratory criteria for APS, for example, lupus anticoagulant (LA), anti-cardiolipin antibodies (aCL), and anti-β2GPI antibodies (aβ2GPI) (12, 13). In our prospective, multicenter, cross-sectional study, anti-β2GPI/HLA-DR antibodies were most frequently detected in women with RPL (22.9%), and approximately one-fifth (19.8%) of women with unexplained RPL tested positive for these autoantibodies (13). Since January 2021, measurements of serum anti-β2GPI/HLA-DR antibody levels were standardized, and the normal range of anti-β2GPI/HLA-DR antibody level (<73.3 U) was determined using sera from 374 healthy controls at the 99th percentile (14). Using the standardized measurement method in our other prospective, multicenter, cross-sectional study, 16.9% of women with RPL, 17.4% of those with hypertensive disorders of pregnancy (HDP), and 15.3% of those with fetal growth restriction (FGR) tested positive for anti-β2GPI/HLA-DR antibody. Furthermore, anti-β2GPI/HLA-DR antibody positivity was found to be a risk factor for RPL (adjusted odds ratio [aOR] 3.3), HDP (aOR 2.7), and FGR (aOR 2.7) (14). A previous study suggested that the aberrant expression of HLA-DR in syncytiotrophoblast were observed in the patients with preeclampsia (PE) but not in healthy control pregnant women (15). According to the results of these studies, it is hypothesized that aberrantly expressed HLA-DR in the placenta forms complexes with β2GPI. anti-β2GPI/HLA-DR antibody can bind to trophoblasts via β2GPI, ultimately leading to autoantibody-induced damage to placental functions.

Therefore, these results demonstrate that anti-β2GPI/HLA-DR antibodies can be a major risk factor for RPL, and therapeutic strategies for RPL associated with anti-β2GPI/HLA-DR antibodies may improve pregnancy outcomes in women suffering from RPL, particularly unexplained RPL. However, no studies have evaluated treatment efficacy in women with RPL and anti-β2GPI/HLA-DR antibody positivity. Because the combination of low-dose aspirin (LDA) and heparin (unfractionated heparin [UFH] or low-molecular-weight heparin [LMWH]) is the standard treatment for pregnant women with APS (16), these therapies may be also effective in women with RPL and anti-β2GPI/HLA-DR antibody positivity.

In this prospective, multicenter, observational cohort study, we aimed to assess the efficacy of LDA and/or heparin therapies in women with RPL and anti-β2GPI/HLA-DR antibody positivity.

## Material and methods

### Patient enrollment

This prospective multicenter observational study was approved by the review boards of five medical centers (Reference no. 190102 at Kobe University Hospital), and all participants provided written informed consent. This study was registered at the University Hospital Medical Network as #000037771. Women with RPL who visited one of the five centers between August 2019 and December 2021 underwent measurements of serum anti-β2GPI/HLA-DR antibody levels and conventional assessments to identify causes and risk factors for RPL. In this study, RPL was defined as the occurrence of two or more pregnancy losses according to the definition of the Japan Society of Obstetrics and Gynecology and the American Society for Reproductive Medicine (17).

The study enrolled women with RPL who tested positive for anti-β2GPI/HLA-DR antibodies and became pregnant. Each attending physician decided the treatment modality for these women, and pregnancy outcomes in women with RPL and anti-β2GPI/HLA-DR antibody positivity were followed up until December 2023.

The following clinical data of all participants were collected: maternal age; gravidity and parity; body mass index (BMI); previous and current maternal complications, including thrombosis and autoimmune diseases; smoking habits; history of assisted reproductive technology (ART); previous or current pregnancy complications; gestational weeks (GWs) at delivery; birth weights; and sex of newborns.

### Clinical checkups to identify causes and risk factors for RPL

To identify causes and potential risk factors for RPL, all participants received checkups including ultrasound examinations to detect uterine malformations, serum tests of thyroid function (e.g., levels of thyroid-stimulating hormone and free thyroxine were measured using an Architect i2000SR Analyzer [Abbott, Tokyo, Japan]), chromosome karyotyping (Special References Laboratories [SRL], Tokyo, Japan) of the peripheral blood from women and their partners, measurements of serum levels of aPL (e.g., LA, Ig G/M aCL, and β2GPI-dependent aCL [aCL/β2GPI, SRL], and IgG/M aβ2GPI [a Quanta Flash Antiphospholipid Assay Panel, Inova Diagnostics, San Diego, USA]), and hemostatic molecular markers (e.g., protein S, protein C, and coagulation factor XII activities [SRL]).

### Method for measuring anti-β2GPI/HLA-DR antibody levels

Serum levels of anti-β2GPI/HLA-DR antibodies (normal, <73.3 U) were measured at HuLA Immune Inc. (Tokyo, Japan), currently Revorf Co., Ltd. (Tokyo, Japan), following previously described standardized methods (14). cDNA was prepared from pooled human peripheral blood mononuclear cells (3H Biomedical, Uppsala, Sweden) and cloned into pME18S or pCAGGS expression vectors. Polyethylenimine Max Reagent (PolyScience, Valley Road Warrington, PA, USA) was used for transient transfection. 293T cells were cotransfected with GFP, β2GPI, and HLA–DRA*01:01 and DRB1*07:01 or with DsRed and HLA–DRA*01:01 and DRB1*07:01 to generate GFP-labeled β2GPI/HLA-DR-expressing cells or DsRed-labeled HLA-DR-expressing cells. Each transfectant was aliquoted (3 × 10^6^ cells/tube) with the cryoprotectant medium (Cell Banker 1 Plus, Takara, Kusatsu, Japan) and stored at −80°C until used. The mean fluorescence intensity of IgG binding to these transfected cells in the sample sera was analyzed using flow cytometry (FACS Lyric, Becton Dickinson, Franklin Lakes, NJ, USA). Both transfected cells were simultaneously incubated with each serum sample in 96-well plates (10^2^-fold dilution, 20 μL, for 30 min) and then incubated for 20 min with the APC-labeled anti-human IgG (Jackson ImmunoResearch, West Grove, PA, USA). Specific IgG binding to the β2GPI/HLA-DR complex was calculated by subtracting the mean fluorescence intensity of IgG binding to cells transfected with HLA-DR alone from cells transfected with both β2GPI and HLA-DR. A serum sample from a woman with RPL and anti-β2GPI/HLA-DR antibody positivity after 10^6^-fold dilution was used as a standard defined as 1,000 units (U) (13). A serum from a woman with APS (12) was used for unit calibration. In each serum sample, anti-β2GPI/HLA-DR antibody levels were calculated from the standard curve, which was generated by measuring specific IgG binding to the β2GPI/HLA-DR complex in serially diluted standard serum (10^2^- to 10^6^-fold dilution). All measurements were performed in duplicate, and mean values were defined as the anti-β2GPI/HLA-DR antibody level of the sample.

### Treatment modality for women with RPL

Treatments for women with RPL were decided according to the risk factors for RPL of each patient. Hyperthyroidism and hypothyroidism, including subclinical hypothyroidism, were treated with antithyroid drugs and levothyroxine, respectively. Women with APS received LDA + UFH. Occasional aPL-positive women with PRL received LDA alone or LDA + UFH. Similarly, women who had low serum activity of coagulation factor XII, protein S, or protein C received LDA alone or LDA + UFH. Therapy with LDA (aspirin: 81–100 mg/day) was initiated prior to conception and was continued until 28 GWs. When UFH was used in conjunction with LDA, a prophylactic dose of UFH (5000 units, twice daily) was commenced as soon as pregnancy was confirmed, and was continued until 36 GWs. Women with unexplained RPL did not receive treatment, or received 5 mg/day of prednisolone (PSL) or a high dose of intravenous immunoglobulin (IVIG) with informed consent. Especially, high-dose IVIG therapies were administered to women who had experienced ≥4 unexplained RPL or who had LDA + UFH-refractory APS.

Each attending physician offered information about treatment options (e.g., no treatment, LDA, LDA + UFH, etc.) to women with RPL and anti-β2GPI/HLA-DR antibody positivity, and they received treatments with informed consent.

### Comparison of pregnancy outcomes in women with RPL and anti-β2GPI/HLA-DR antibody positivity according to differences in treatment modality

All pregnancies in women with RPL and anti-β2GPI/HLA-DR antibody positivity were divided into two groups according to the treatment options: (1) the LDA/UFH group that received LDA and/or UFH and (2) the non-LDA/non-UFH group treated without LDA or UFH. The live birth rates among all the participants and pregnancy complication rates among participants who had live births were compared between the two groups. Pregnancy complications included PE and preterm delivery before 34 GWs (PD ≤34 GWs) caused by placental insufficiency (e.g., FGR) according to the clinical symptoms in the diagnostic criteria for APS. PE was defined as hypertension during pregnancy accompanied by proteinuria, organ damage, or uteroplacental dysfunction and identified after 20 GWs. Hypertension was defined as a maternal systolic blood pressure (BP) of ≥140 mmHg and/or diastolic BP of ≥90 mmHg on two or more occasions at least 4 h apart. FGR was defined as an estimated fetal body weight ≤ the mean −1.5 standard deviation for GWs.

Pregnancies that ended in miscarriages because of fetal chromosome abnormalities and those that ended in fetal deaths due to obvious causes (e.g., severe fetal infection, placental abruption, and umbilical cord prolapse) were excluded from the analysis.

### Statistics

Differences in clinical characteristics, live birth rates, and pregnancy complication rates between the LDA/UFH and non-LDA/non-UFH groups were analyzed using the Mann–Whitney *U* and Fisher exact tests. Statistical significance was set at a *p-*value <0.05. All statistical analyses were performed using EZR (Saitama Medical Center, Jichi Medical University, Saitama, Japan), a graphical user interface for R (The R Foundation for Statistical Computing, Vienna, Austria).

## Results

### Study population

**Figure 1:**
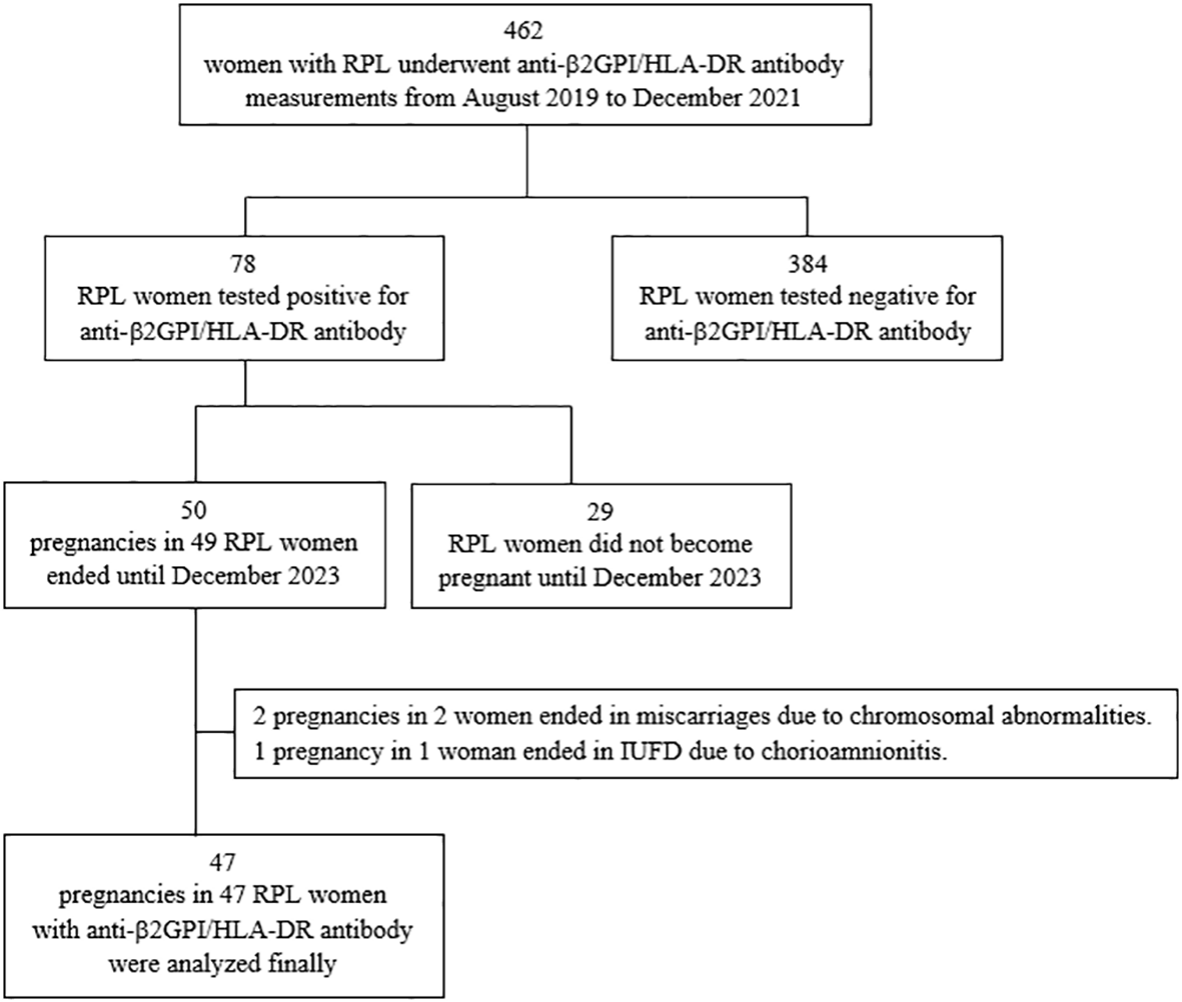
Flow diagram of the participants and their pregnancies included in the final analysis. RPL, recurrent pregnancy loss; β2GPI, β2-glycoprotein I; HLA, human leukocyte antigen; IUFD, intrauterine fetal death.

From August 2019 to December 2021, serum anti-β2GPI/HLA-DR antibody levels (normal, <73.3 U) were measured in 462 women with RPL, and 78 (16.9%) of them tested positive for anti-β2GPI/HLA-DR autoantibodies as described previously (14). Between August 2019 and December 2023, 49 (62.3%) of the 78 women with RPL and anti-β2GPI/HLA-DR antibody positivity experienced 50 pregnancies, and all these outcomes were assessed. Two pregnancies ending in miscarriages because of chromosomal abnormalities and one in intrauterine fetal death at 24 GWs due to chorioamnionitis after preterm rupture of the membranes were excluded from the analysis. During the study period, one woman had two pregnancies, and one of the two had chromosomal abnormalities. Therefore, 47 pregnancies in the 47 women with RPL and anti-β2GPI/HLA-DR antibody positivity were included in the final analysis (**Figure 1**).

Among the 47 participants in the final analysis, 6 did not receive treatment. The remaining 41 participants received the following treatments: LDA alone (n = 12), LDA + IVIG (n = 1), LDA + UFH (n = 19), LDA + UFH + PSL (n = 4), LDA + UFH + IVIG (n = 2), LDA + UFH + IVIG + PSL (n = 1), and PSL alone (n = 2). Therefore, 39 participants received treatments including LDA or UFH (LDA/UFH group), and the remaining 8 did not receive LDA or UFH (non-LDA/non-UFH group) but with PSL (n = 2) and no treatment (n = 6). The clinical characteristics and pregnancy outcomes in all 47 participants consisting of the LDA/UFH (n = 39) and non-LDA/non-UFH (n = 8) groups are shown in **Table 1**.

**Table 1:** Treatment modalities, clinical characteristics, and pregnancy outcomes in all 47 participants included in the final analysis.

No.	Treatments	Anti-β2GPI/ HLA-DR antibody levels (U)	Age (y)	BMI (kg/m^2^)	Auto-immune diseases	History of thrombosis	Gravidity (no.)	Parity (no.)	History of miscarriages (no.)	History of stillbirths at 22GWs or later (no.)	Pregnancy following ART	Risk factors for RPL	Pregnancy outcome (GWs)	Pregnancy complications
1	LDA	216.7	37	27.4	−	−	5	2	0	2	No	Low PS activity	Live birth (38)	−
2	LDA	86.3	30	25.6	−	−	3	0	2	0	No	Unexplained	Live birth (39)	−
3	LDA	78.9	30	20.2	−	−	4	0	3	0	No	Low PS activity	Live birth (41)	−
4	LDA	116.0	37	19.4	−	−	3	0	1	1	Yes	Low FXII activity	Live birth (35)	PD
5	LDA	81.6	36	23.7	−	−	3	0	2	0	No	Unexplained	Live birth (40)	−
6	LDA	198.0	39	17.5	−	−	3	0	2	0	No	Thyroid dysfunction	Live birth (39)	−
7	LDA	301.3	37	20.2	−	−	4	0	3	0	No	Unexplained	Live birth (39)	LGA
8	LDA	78.4	34	20.8	−	−	3	0	2	0	No	Unexplained	Live birth (40)	−
9	LDA	121.2	30	19.1	−	−	3	0	2	0	Yes	Unexplained	Live birth (40)	−
10	LDA	98.9	33	20.3	−	−	4	0	3	0	No	Thyroid dysfunction, Low PC activity	Live birth (37)	−
11	LDA	165.8	27	20.1	−	−	3	0	2	0	Yes	Tyroid dysfunction, aPL positive	Live birth (38)	−
12	LDA	173.4	32	22.6	−	−	3	0	1	1	No	Unexplained	Live birth (38)	−
13	LDA + IVIG	174.7	31	19.8	−	−	7	0	7	0	No	Unexplained	Live birth (37)	−
14	LDA + UFH	100.9	33	23.2	−	−	4	1	2	0	No	aPL positive Low PC activity	Live birth (40)	−
15	LDA + UFH	136.9	35	20.4	−	−	4	0	3	0	Yes	Unexplained	Live birth (40)	LGA
16	LDA + UFH	142.3	36	21.8	−	−	4	0	3	0	No	Unexplained	Live birth (40)	−
17	LDA + UFH	83.7	39	21.8	−	−	4	0	3	0	No	Thyroid dysfunction	Live birth (40)	−
18	LDA + UFH	85.8	35	20.8	Sjs	−	3	0	2	0	No	Unexplained	Live birth (39)	−
19	LDA + UFH	106.0	33	21.4	−	−	4	1	2	0	No	Uterine malformation	Live birth (36)	PD
20	LDA + UFH	91.6	28	19.1	−	−	4	0	3	0	No	Unexplained	Live birth (38)	−
21	LDA + UFH	199.3	35	21.2	−	−	3	0	2	0	No	Unexplained	Live birth (38)	−
22	LDA + UFH	108.3	29	27.1	−	−	5	1	3	0	No	aPL positive Low PC activity	Live birth (34)	HPD (PE), PD
23	LDA + UFH	1235.5	40	24.5	−	−	4	1	2	0	Yes	aPL positive	Live birth (40)	−
24	LDA + UFH	130.9	43	18.4	−	−	4	1	2	0	Yes	Low PS activity	Live birth (36)	PD
25	LDA + UFH	90.8	34	21.6	−	−	3	0	2	0	Yes	Unexplained	Live birth (39)	−
26	LDA + UFH	85.2	30	20.3	APS	−	4	0	3	0	No	aPL positive	Live birth (38)	−
27	LDA + UFH	139.3	27	25.8	−	−	3	0	2	0	No	Low FXII activity	Live birth (40)	−
28	LDA + UFH	96.7	42	17.8	−	−	4	0	3	0	No	Low PS activity	Live birth (41)	−
29	LDA + UFH	76.2	34	18.8	−	−	3	0	2	0	Yes	Thyroid dysfunction aPL postive	Live birth (34)	HDP (PE), PD
30	LDA + UFH	249.0	29	21.3	APS	−	3	0	2	0	No	aPL positive Low FXII activity	Live birth (38)	−
31	LDA + UFH	664.9	33	18.8	−	−	6	0	5	0	Yes	aPL positive	Miscarriage (7)	
32	LDA + UFH	96.7	40	23.6	−	−	3	0	2	0	Yes	aPL positive	Miscarriage (7)	
33	LDA + UFH + PSL	78.0	33	18.3	−	−	4	0	2	0	No	Low PC activity, Low PS activity	Live birth (38)	−
34	LDA + UFH + PSL	73.5	33	20.9	APS	−	5	0	3	0	No	aPL positive, Low FXII activity, Low PS activity	Live birth (36)	PD
35	LDA + UFH + PSL	124.7	43	22.6	SLE, APS	−	4	1	0	2	No	aPL positive	Live birth (35)	PD
36	LDA + UFH + PSL	86.0	32	19.8	DM	−	4	0	3	0	No	Unexplained	Miscarriage (9)	
37	LDA + UFH + IVIG	194.0	48	19.1	−	−	3	0	2	0	Yes	Thyroid dysfunction	Live birth (36)	PD
38	LDA + UFH + IVIG	104.6	29	19.9	−	−	6	0	5	0	No	Low PS activity	Miscarriage (8)	
39	LDA + UFH + IVIG + PSL	164.0	37	29.5	−	+	3	0	2	0	Yes	aPL positive	Miscarriage (7)	
40	None	124.7	31	20.8	−	−	5	1	2	0	No	Unexplained	Live birth (38)	−
41	None	123.1	40	22.8	Graves’ disease	−	5	1	3	0	No	Thyroid dysfunction	Live birth (35)	HDP (PE), FGR, PD, LGA
42	None	85.3	34	23.4	−	−	4	1	2	0	Yes	Unexplained	Live birth (39)	HDP (CH)
43	None	93.1	32	19.6	−	−	3	0	2	0	No	Unexplained	Live birth (33)	HDP (PE), PD
44	None	166.8	35	21.9	SLE	−	5	1	3	0	Yes	Unexplained	Miscarriage (7)	
45	None	79.7	37	27.1	−	−	5	2	1	1	No	Unexplained	Miscarriage (14)	
46	5mg/day of PSL	160.9	35	29.7	−	−	3	2	2	0	No	Unexplained	Miscarriage (6)	
47	5mg/day of PSL	163.6	27	19.8	−	−	3	2	2	0	No	Unexplained	Miscarriage (8)	

β2GPI, β2-glycoprotein I; HLA, human leukocyte antigen; RPL, recurrent pregnancy loss; LDA, low-dose aspirin; UFH, unfractionated heparin; BMI, body mass index; GWs, gestational weeks; ART, assisted reproductive technology; PS, protein S; FXII, factor XII; PD, preterm delivery; LGA, light-for-gestational age; IVIG, intravenous immunoglobulin; PC, protein C; Sjs, Sjögren's syndrome; HPD, hypertensive disorders of pregnancy; PE, preeclampsia, CH, chronic hypertension; APS, antiphospholipid syndrome; aPL, antiphospholipid antibody; PSL, prednisolone; SLE, systemic lupus erythematosus; DM, dermatomyositis.

### Clinical characteristics of the participants

**Table 2** shows the clinical characteristics of all 47 participants in the final analysis and the comparison between the LDA/UFH (n = 39) and non-LDA/non-UFH (n = 8) groups.

**Table 2:** Clinical characteristics of participants enrolled in the final analysis.

	Total n=47	LDA/UFH n=39	Non-LDA/non-UFH n=8	*p*-value
Age, years	34 (27–48)	34 (27–48)	35 (27–40)	1.0
BMI, kg/m^2^	20.8 (17.5–29.7)	20.8 (17.5–29.5)	22.3 (19.6–29.7)	0.2
Gravidity, no.	4 (3–7)	4 (3–7)	5 (3–5)	0.3
Parity, no.	0 (0–2)	0 (0–2)	1 (0–2)	5.0 x10^-5^
History of miscarriages, no.	2 (0–7)	2 (0–7)	2 (1–3)	0.5
History of stillbirths at 22 GWs or later, no.	0 (0–2)	0 (0–2)	0 (0–1)	0.9
Pregnancy following ART	14 (29.8%)	12 (30.8%)	2 (25.0%)	1.0
Autoimmune diseases	8 (17.0%)	6 (15.4%)	2 (25.0%)	0.6
History of thrombosis	1 (2.1%)	1 (2.6%)	0 (0.0%)	1.0
Unexplained RPL	21 (44.7%)	14 (35.9%)	7 (87.5%)	0.02
Anti-β2GPI/HLA-DR antibody value, U	116.0 (73.5–1235.5)	108.3 (73.5–1235.5)	123.9 (79.7–166.8)	0.9

Data are expressed as the median (range) or number (percentage). LDA, low-dose aspirin; UFH, unfractionated heparin; BMI, body mass index; No, number; GWs, gestational weeks; ART, assisted reproductive technology; RPL, recurrent pregnancy loss; β2GPI, β2-glycoprotein I; HLA, human leukocyte antigen; U, unit.

The number of parity and proportion of participants with unexplained RPL in the LDA/UFH group were significantly lower than those in the non-LDA/non-UFH group. Age, BMI, gravidity, number of previous miscarriages and stillbirths at 22 GWs or later, proportion of patients who had pregnancies following ART, autoimmune diseases, and history of thrombosis, or titers of anti- β2GPI/HLA-DR antibodies were not significantly different between the groups.

### Treatment modality and pregnancy outcomes in women with RPL and anti-β2GPI/HLA-DR antibody positivity

**Table 3** shows the treatment modality and pregnancy outcomes in women with RPL and anti-β2GPI/HLA-DR antibody positivity according to background and characteristics. In this study, 34 (87.2%) of the 39 patients in the LDA/UFH group and 4 (50.0%) of the 8 in the non-LDA/non-UFH group had live births. In addition, 2 (5.9%) of 34 participants who had live births in the LDA/UFH group and 2 (50.0%) of 4 who had live births in the non-LDA/non-UFH group had pregnancy complications.

**Table 3:** Treatment modality and pregnancy outcomes in RPL women with anti-β2GPI/HLA-DR antibody.

Treatment modality	No.of pregnancies in women with anti-β2GPI/ HLA-DR Ab (+)	Percentages of live birth (number ^a)^)				Percentages of pregnancy complications (number ^b)^)			
		Women with anti-β2GPI/ HLA-DR Ab (+) n=47	Women with anti-β2GPI/ HLA-DR Ab (+) and aPL (-) n=35	Women with anti-β2GPI/ HLA-DR Ab (+) and aPL (+) n=12	Women with anti- β2GPI/ HLA-DR Ab (+) and unexplained RPL n=21	Women with anti-β2GPI/ HLA-DR Ab (+) n=38	Women with anti-β2GPI/ HLA-DR Ab (+) and aPL (-) n=29	Women with anti-β2GPI/ HLA-DR Ab (+) and aPL (+) n=9	Women with anti-β2GPI/ HLA-DR Ab (+) and unexplained RPL n=16
LDA/UFH group									
LDA	12	100 (12/12)	100 (11/11)	100 (1/1)	100 (6/6)	0 (0/12)	0 (0/11)	0 (0/1)	0 (0/6)
LDA + IVIG	1	100 (1/1)	100 (1/1)	N.A.	100 (1/1)	0 (0/1)	0 (0/1)	N.A.	0 (0/1)
LDA + UFH	19	89.5 (17/19)	100 (11 /11)	75 (6/8)	100 (6/6)	11.8 (2/17)	0 (0/11)	33.3 (2/6)	0 (0/6)
LDA+UFH + PSL	4	75 (3/4)	50 (1/2)	100 (2/2)	0 (0/1)	0 (0/3)	0 (0/1)	0 (0/2)	N.A.
LDA+UFH + IVIG	2	50 (1/2)	50 (1/2)	N.A.	N.A.	0 (0/1)	0 (0/1)	N.A.	N.A.
LDA+UFH + IVIG+PSL	1	0 (0/1)	N.A.	0 (0/1)	N.A.	N.A.	N.A.	N.A.	N.A.
Total	39	87.2 (34/39)	92.6 (25/27)	75 (9/12)	92.9 (13/14)	5.9 (2/34)	0 (0/25)	22.2 (2/9)	0 (0/13)
Non-LDA/ non-UFH group									
None	6	66.7 (4/6)	66.7 (4/6)	N.A.	60 (3/5)	50 (2/4)	50 (2/4)	N.A.	33.3 (1/3)
5 mg/day of PSL	2	0 (0/2)	0 (0/2)	N.A.	0 (0/2)	N.A.	N.A.	N.A.	N.A.
Total	8	50 (4/8)	50 (4/8)	N.A.	42.9 (3/7)	50 (2/4)	50 (2/4)	N.A.	33.3 (1/3)

^a)^ Data are expressed as number of pregnancies ending in live births in each population / total number of pregnancies in each population (percentage). ^b)^ Data are expressed as number of pregnancies complicated by preeclampsia or preterm delivery before 34 gestational weeks due to placental insufficiency among the participants who had live births in each population / total number of pregnancies among the participants who had live births in each population (percentage).

β2GPI, β2-glycoprotein I; HLA, human leukocyte antigen; antibody, Ab; aPL, antiphospholipid antibody; RPL, recurrent pregnancy loss; LDA, low-dose aspirin; UFH, unfractionated heparin; PSL, prednisolone; IVIG, intravenous immunoglobulin; N.A., not applicable

### Comparisons of live birth rates and pregnancy complication rates between the LDA/UFH and non-LDA/non-UFH groups

The comparisons of live birth rates and pregnancy complication rates between the two groups in different categories are shown in **Tables 4** and **5**, respectively.

**Table 4:** The comparisons of the live birth rate between the LDA/UFH group and the non-LDA/non-UFH group in different categories.

Categories of women with RPL	No. of patients	Live birth rate in the LDA/UFH group	Live birth rate in the non-LDA/ non-UFH group	*p*-value
Anti-β2GPI/HLA-DR antibody-positive	47	87.2% (34/39)	50.0% (4/8)	0.03
Anti-β2GPI/HLA-DR antibody (+) and aPL (-)	35	92.6% (25/27)	50.0% (4/8)	0.02
Anti-β2GPI/HLA-DR antibody (+) and aPL (+)	12	75.0% (9/12)	N.A.	N.D.
Anti-β2GPI/HLA-DR antibody (+) and unexplained RPL	21	92.9% (13/14)	42.9% (3/7)	0.03

Data are expressed as percentage (number of pregnancies ending in live births in each population / total number of pregnancies in each population).

LDA, low-dose aspirin; UFH, unfractionated heparin; β2GPI, β2-glycoprotein I; HLA, human leukocyte antigen; RPL, recurrent pregnancy loss; N.A., not applicable; N.D., not determined.

**Table 5:** The comparisons of the pregnancy complication rate between the LDA/UFH group and the non-LDA/non-UFH group in different categories.

Categories of RPL women who had live births	No. of patients	Pregnancy complication rate in the LDA/UFH group	Pregnancy complication rate in the non-LDA/ non-UFH group	*p*-value
Anti-β2GPI/HLA-DR antibody-positive	38	5.9% (2/34)	50.0% (2/4)	0.048
Anti-β2GPI/HLA-DR antibody (+) and aPL (-)	29	0% (0/25)	50.0% (2/4)	0.01
Anti-β2GPI/HLA-DR antibody (+) and aPL (+)	9	22.2% (2/9)	N.A.	N.D.
Anti-β2GPI/HLA-DR antibody (+) and unexplained RPL	16	0% (0/13)	33.3% (1/3)	0.2

Data are expressed as percentage (number of pregnancies complicated by preeclampsia or preterm delivery before 34 gestational weeks due to placental insufficiency among the participants who had live births in each population / total number of pregnancies among the participants who had live births in each population).

APS, antiphospholipid syndrome; LDA, low-dose aspirin; UFH, unfractionated heparin; β2GPI, β2-glycoprotein I; HLA, human leukocyte antigen; RPL, recurrent pregnancy loss; N.A., not applicable; N.D., not determined.

Among the 47 women with PRL and anti-β2GPI/HLA-DR antibody positivity, the live birth rate in the LDA/UFH group was significantly higher than that in the non-LDA/non-UFH group (87.2% vs 50.0%, *p* = 0.03). In addition, among the 38 women with RPL and anti-β2GPI/HLA-DR antibody positivity who had live births, the pregnancy complication rate in the LDA/UFH group was significantly lower than that in the non-LDA/non-UFH group (5.9% vs 50.0%, *p* = 0.048).

In the 38 women with RPL who tested positive for anti-β2GPI/HLA-DR antibodies but negative for aPLs, the live birth rate in the LDA/UFH group was significantly higher than that in the non-LDA/non-UFH group (92.6% vs 50.0%, *p* = 0.02). Among the 29 women with RPL and anti-β2GPI/HLA-DR antibody positivity but negative for aPLs who had live births, the pregnancy complication rate in the LDA/UFH group was significantly lower than that in the non-LDA/non-UFH group (0% vs 50.0%, *p* = 0.01).

The live birth and pregnancy complication rates of RPL women who tested positive for both anti-β2GPI/HLA-DR antibodies and aPLs could not be compared because all patients in this population received treatments including LDA or UFH.

Among the 21 women with unexplained RPL and anti-β2GPI/HLA-DR antibody positivity, the live birth rate in the LDA/UFH group was significantly higher than that in the non-LDA/non-UFH group (92.9% vs 42.9%, *p* = 0.03). Among the 16 women with unexplained RPL and anti-β2GPI/HLA-DR antibody positivity who had live births, the pregnancy complication rate in the LDA/UFH group was lower than that in the non-LDA/non-UFH group, but the difference was not significant (0% vs 33.3%, *p* = 0.2).

## Discussion

This study, for the first time, demonstrated that LDA and/or UFH therapies in women with RPL and aβ2GPI/HLA-DR antibody positivity are effective in improving pregnancy outcomes compared with treatments without LDA or UFH. The combination therapy of LDA and heparin (UFH or LMWH) is the standard treatment for pregnant women with APS (16). The use of LDA or heparin in women with unexplained RPL was not recommended because no evidence confirms the improvement of live birth rates (16). β2GPI/HLA-DR complexes expose not only the epitopes in β2GPI, which can be recognized by aPLs that met the laboratory criteria for APS diagnosis, but also unique epitopes, which can be recognized only by anti-β2GPI/HLA-DR antibodies (12). In this study, 13 (92.9%) of the 14 women with unexplained RPL and anti-β2GPI/HLA-DR antibody positivity had live births after LDA and/or UFH therapies. As women with unexplained RPL possibly include those with seronegative APS, LDA and/or heparin therapies can be recommended in women with unexplained RPL and anti-β2GPI/HLA-DR antibody positivity.

Two (50.0%) of 4 women with anti-β2GPI/HLA-DR antibody positivity who had live births without any treatment experienced pregnancy complications (1 case with PE [case 43 in **Table 1**] and 1 with PE and PD ≤34 GWs because of FGR [case 41 in **Table 1**]). In contrast, 2 (5.9%) of 34 women with anti-β2GPI/HLA-DR antibody positivity who had live births treated with LDA and/or UFH therapies experienced pregnancy complications (2 cases with PE [cases 22 and 29 in **Table 1**]). The rate of pregnancy complications in the LDA/UFH group was significantly lower than that in the non-LDA/non-UFH group (*p* = 0.048). A recent study demonstrated that anti-β2GPI/HLA-DR antibody positivity was a significant risk factor for HDP and FGR (14). In addition to RPL, unexplained fetal death beyond 10 GWs, premature births of normal neonates before 34 GWs due to eclampsia, severe eclampsia or recognized features of placental insufficiency are pregnancy morbidities included in the clinical criteria for APS diagnosis (5). LDA and/or heparin therapies during pregnancy in women with RPL and anti-β2GPI/HLA-DR antibody positivity may be effective in preventing not only miscarriages but also APS-associated pregnancy complications.

The aPLs can bind to trophoblast and endometrial endothelial cells via β2GPI, which itself binds to phospholipids expressed on these cells, and then aPLs interfere with both trophoblast functions and with the differentiation of endometrial endothelial cells (11). In animal models of aPL‐induced fetal loss, acute inflammatory events evoke aPL‐mediated placental damage through the activation of pro‐inflammatory mediators, such as complement, tumor necrosis factor‐α (TNF‐α), and chemokines (6, 10, 19). On the other hand, LMWH enhances the invasiveness of trophoblasts and the differentiation of endometrial endothelial cells by inhibiting the binding of aPLs to these cells. In addition, LMWH promotes the angiogenesis of endometrial endothelial cells, the activities of NF-κB and/or STAT-3, and matrix metalloproteinase, as well as the secretion of VEGF, by counteracting the suppressive activities of aPLs (18). The both UFH and LMWH prevent obstetric complications in APS patients by inhibiting complement activation, rather than through their anticoagulation effects (20). It was also found that interleukin-3 (IL-3) could restore the placental functions (21), and a potentiator of IL-3 production, such as LDA, could prevent fetal loss (22, 23). These mechanisms may be involved in the effectiveness of LDA and UFH therapies in preventing miscarriages and pregnancy complications in women with RPL and anti-β2GPI/HLA-DR antibody positivity.

Women with APS of multi-aPLs positivity, particularly women with triple positivity, had lower live birth rates and higher incidence rates of PE and FGR than those with single aPL positivity (24). In this study, one woman who tested positive for LA, IgG aCL, IgM aCL, and aCL/β2GPI had a miscarriage despite receiving LDA + UFH + IVIG + PSL (case 39 in **Table 1**). The live birth rates in women with APS, anti-β2GPI/HLA-DR antibody positivity, and LDA and/or UFH (75.0% [9/12]) therapy were relatively lower than the rates in those without aPL (92.6%, [25/27]) (*p* = 0.16). The 20%–30% of pregnant women with APS are refractory to combination therapy of LDA and heparin (25). Anti-β2GPI/HLA-DR antibody positivity may increase resistance to LDA and/or heparin therapies in women with RPL and APS. IVIG, hydroxychloroquine, plasma exchange, and pravastatin could be effective second-line therapies for pregnant women with APS refractory to the combination therapy (26, 27). These second-line therapies can be considered for women with RPL and anti-β2GPI/HLA-DR antibody positivity refractory to the combination therapy of LDA and heparin (cases 31, 32, 36, 38, and 39 in **Table 1**).

The ESHRE guideline did not recommend glucocorticoids as a treatment of unexplained RPL (16). All women with anti-β2GPI/HLA-DR antibody positivity and unexplained RPL who received 5 mg/day of PSL alone had miscarriages (cases 46 and 47 in **Table 1**). PSL therapy may be not effective for women with RPL and anti-β2GPI/HLA-DR antibody positivity. However, two of four women with RPL and anti-β2GPI/HLA-DR antibody positivity who received high-dose IVIG therapy had live births (cases 13 and 37 in **Table 1**). Since a randomized controlled trial (RCT) demonstrated that high-dose IVIG therapy in early pregnancy improves the live birth rate in women with ≥4 unexplained RPL (28), the IVIG therapy may be effective in women with unexplained RPL and anti-β2GPI/HLA-DR antibody positivity.

A recent study has discovered that anti-β2GPI/HLA-DR antibodies are involved in infertility, endometriosis, and recurrent implantation failure. β2GPI and HLA-DR are expressed coordinately in epithelial cells of endometriotic lesions by immunofluorescent staining, suggesting implantation failure caused by endometrial inflammation (29). LDA and/or heparin therapy may be also effective treatment for infertility and recurrent implantation failure in women with anti-β2GPI/HLA-DR antibody positivity. More recently, anti-β2GPI/HLA-DR antibody positivity with a cutoff of 172.359 U has been discovered to be a risk for arterial thrombosis in women with systemic rheumatic diseases (OR 5.13) (30). Therefore, anti-β2GPI/HLA-DR antibodies appear to be also involved in the pathology of thrombotic APS which is clinically distinguished from obstetric APS.

This study demonstrated that LDA and/or heparin therapy may be effective in improving live birth rates and preventing pregnancy complications among women with RPL and anti-β2GPI/HLA-DR antibody positivity. The results of this study provide useful information for clinical practitioners. However, this study had several limitations. First, the total number of participants was relatively small (n=47), and the number of participants in each subgroup was also small (the maximum was 19 in the group of women who received LDA+UFH). Second, there was heterogeneity among the subgroups based on treatment modalities. For instance, some patients received LDA alone, while others received LDA+UFH+IVIG, etc. Third, the study was not a RCT. Fourth, this study included women with who tested positive for both anti-β2GPI/HLA-DR antibody and aPLs. It was unclear whether the results were more strongly influenced by the presence of aPLs than by anti-β2GPI/HLA-DR antibody. Thus, RCTs that include a large number of cases are needed. In addition, administrations of LMWH to pregnant women have been off-label in Japan; therefore, we could not evaluate the efficacy of LMWH in women with RPL and anti-β2GPI/HLA-DR antibody positivity.

## Data Availability

The raw data supporting the conclusions of this article will be made available by the authors, without undue reservation.
